# Effects of Lentil Genotype on the Colonization of Beneficial *Trichoderma* Species and Biocontrol of *Aphanomyces* Root Rot

**DOI:** 10.3390/microorganisms8091290

**Published:** 2020-08-24

**Authors:** Navid Bazghaleh, Pratibha Prashar, Sheridan Woo, Albert Vandenberg

**Affiliations:** 1 Department of Plant Sciences, University of Saskatchewan, Saskatoon, SK S7N5A8, Canada; pratibha.prashar@usask.ca; 2 Department of Pharmacy, University of Naples Federico II, 80131 Naples, Italy; woo@unina.it; 3 National Research Council, Institute for Sustainable Plant Protection, 80055 Portici, Italy

**Keywords:** lentil genotype, *Trichoderma*, *Aphanomyces euteiches*, multipartite interactions, root rot, symbiosis

## Abstract

*Trichoderma* species are opportunistic plant symbionts that are common in the root and rhizosphere ecosystems. Many *Trichoderma* species may enhance plant growth, nutrient acquisition, and disease resistance, and for these reasons, they are widely used in agriculture as biofertilizers or biocontrol agents. Host plant genotype and other microorganisms, such as root pathogens, may influence the efficacy of *Trichoderma* inoculants. *Aphanomyces euteiches* is an important soil-borne oomycete in western Canada that causes root rot in legume crops such as lentil and pea, and there is not yet any significantly resistant varieties or effective treatments available to control the disease. In this study, the composition of root-associated fungal communities and the abundance of *Trichoderma* species, *T. harzianum* strain T-22 and *T. virens* strain G41, was determined in the roots of eight *Lens* genotypes based on internal transcribed spacer (ITS) Illumina MiSeq paired-end sequencing, both in the presence and the absence of the root rot pathogen *Aphanomyces euteiches*. Biocontrol effects of *T. harzianum* on *A. euteiches* was also examined. Significant genotypic variations were observed in the composition of root-associated fungal communities and the abundance of the different *Trichoderma* species in the lentil roots. The presence of *A. euteiches* altered the composition of *Trichoderma* found associated to the lentil genotypes. Biocontrol of *A. euteiches* by *T. harzianum* T22 species was observed in vitro and positive correlations between the abundance of *Trichoderma* and plant root and shoot biomass were observed in vivo. These findings revealed that lentil genotype and infection by the phytopathogen *A. euteiches* greatly influenced the colonization of root-associated fungi and the abundance of the *Trichoderma* species, as well as the effect on plant growth promotion. The multipartite interactions observed among lentil genotypes, *Trichoderma* species and *A. euteiches* suggest possibilities to select compatible host-beneficial microbe combinations in lentil breeding programs and to develop application strategies to harness the beneficial effects of *Trichoderma* inoculants in sustainable crop production systems.

## 1. Introduction

The role of beneficial microorganisms in plant growth and development has been confirmed and reasonable commercial success achieved for given crop-microbe combinations [1,2]. These microbes may facilitate nutrient acquisition, modulate hormone signaling, diminish the adverse effects of biotic and abiotic stresses, and have great potential to reduce the inputs of chemical fertilizers and pesticides [3,4,5,6,7]. However, a major constraint to the efficiency of these biological products is their variable performance under different field conditions or on diverse crops. In addition, the microorganisms do not perform individually, in isolated conditions within the plant microbiome, but form multipartite networks with other microbes including mutualists, antagonists and pathogens [8,9,10]. Understanding the extreme complexity of the multipartite interactions is important for exploiting these associations and improving plant health and sustainable crop production [11]. Genotypic compatibility between the host plant and the microbial strain is a key element towards establishing an efficient symbiotic association [12,13], which has been reported for both bacterial and fungal endophytes [14,15,16,17]. Plant roots exude a wide array of bioactive metabolites and nutrients that may recruit specific microorganisms from the diversity of species present in the soil [18,19,20,21]. Furthermore, the composition and patterns of the metabolites released in the root exudates can change under different biotic and abiotic stress conditions, and may function to attract specific plant beneficial species, including *Trichoderma* [22].

Plant diseases are a main factor responsible for crop losses, accounting for up to 40% reduction in global agricultural production [23]. The management of diseases has become even more challenging in recent times because of the increasingly unpredictable climatic conditions [24], the emergence of new pathogens [25], and a depletion in biodiversity and agro-ecosystem quality [26]. To develop sustainable strategies for disease management and utilize the services provided by agro-ecosystems, plant breeding programs must consider incorporating the potential of the root-associated microorganisms of the plant root microbiome, the so-called ‘extended plant phenotype’, in order to select the efficient crop genotypes that can be cultivated in these conditions.

*Lens* species represent a reservoir of diverse plant phenotypes and genotypes. Lentil (*Lens culinaris*) is a cool season legume and is an expanding food source globally due to its rich protein and mineral content [27]. As a legume, lentil incorporates atmospheric nitrogen into the soil mainly through biological fixation with its associated root symbiotic bacteria, thus influencing and promoting the diversity of soil microbial communities [28]. Canada is the largest producer of lentils contributing 41% of the total global production (FAO, 2017).

*Aphanomyces euteiches* has recently emerged as a challenging root pathogen in western Canada, that is not limited to specific soil or land use types [29]. Root rot diseases caused by this soil-borne oomycete fungus results in substantial losses in pulse crop production, especially under environmental conditions that promote the pathogen growth and development [30,31,32]. Infection results in symptoms of brown-stained roots, generally similar among all infected legumes, leading to poor growth and death of plants. The fungus is adapted to wet soil conditions, and persistent cycles of wet weather in recent years have caused changes in field conditions that have resulted in significant problems with Aphanomyces root rot (ARR) in western Canada, particularly affecting lentil- and pea-growing regions. To date there are no effective control measures for ARR [31,33]. The best approach for the control of ARR could be prevention, sanitation (removal of infected plant material), use of resistant varieties, and improved management practices including crop rotation, promotion of healthy plant growth, and better soil drainage.

Fungicides were not shown to be effective for the control of ARR, however, a recent study has suggested that a combination of partial host resistance and fungicidal treatment may be provide an opportunity to manage ARR [34]. Without effective chemicals to reduce inoculum levels, and without the availability of resistant varieties, it is recommended that the length of time between the rotations of susceptible legumes should be extended to greater than 4 years [33,35]. A major problem for the disease control of ARR is the persistence of up to 10 years of oospores that infest the soil, therefore the 4 year time frame may not be sufficiently long enough to reduce the pathogen populations in the soil. Alternative methods to control ARR in pulse crop production include the use of biological control agents (BCAs). These have a natural arsenal of compounds that are potentially useful for reducing pathogen populations and/or enhancing plant growth. A consideration for augmenting disease resistance could be the use of a combination of treatments, by using BCAs with crop genotypes exhibiting varying levels of pathogen susceptibility–tolerance.

Many *Trichoderma* species have long been recognized as bio-fertilizers, bio-pesticides, plant growth enhancers and stimulants of natural resistance, and there are more than 250 commercial agricultural products containing *Trichoderma* [36]. Depending upon the strain, *Trichoderma* can provide advantages for plant health improvement using various mechanisms such as facilitating nutrient and water acquisition, antagonizing microbial pathogens, and stimulating root growth [37,38,39]. These products are used in greenhouses, nurseries, fields, orchards, and hydroponic systems for nursery, horticultural, fruit-forestry trees and ornamental crops. Biological products containing the active ingredients *T. harzianum* strain T22 and *T. virens* strain G41 are registered for commercial use in Canada. However, there is limited evidence on how this pathogen may alter the composition of the root-associated fungi and the efficacy of potentially beneficial microorganisms *Trichoderma* species. This study hypothesized that lentil genotypes may modify the composition of fungal communities associated with roots. Furthermore, biotic stress, such as the presence of the oomycete pathogen *A. euteiches,* may also influence the abundance of *Trichoderma* spp. in the rhizosphere. To confirm the potential inhibition of *A. euteiches* by *Trichoderma* spp., biocontrol tests were conducted in vitro and in vivo.

## 2. Materials and Methods

### 2.1. Microbial Isolates and Culture Conditions

The oomycete pathogen *Aphanomyces euteiches* used in this study was isolated from lentil plants grown in Saskatchewan (isolate AE1), and obtained from the Pulse Pathology Laboratory, University of Saskatchewan, Canada. Two commercial *Trichoderma* inoculants RootShield^®^ (RS) and RootShield^®^ Plus (RSP) were provided in powder formulations (BioWorks Inc., Victor, NY, USA). RootShield^®^ included spores of *Trichoderma harzianum* strain T22 with the minimum concentration of 1.0 × 10^7^ cfu/g dry weight. RootShield^®^ Plus (RSP) included both the spores of *Trichoderma harzianum* strain strain T-22 as in RS, plus *Trichoderma virens* strain G41 with the minimum concentration of 5.3 × 10^6^ cfu/g dry weight. These biological products for agriculture are currently used for crop protection and the enhancement of productivity in commercial fields.

### 2.2. Plant Material and Experiment Design

Eight lentil genotypes representing wild and cultivated species belonging to three gene pools were used in this study (Table 1). Seed was obtained from the working collection of Crop Development Centre (CDC), University of Saskatchewan, Canada. The seeds were scarified using a blade, surface sterilized by immersion in 1% (*v*/*v*) sodium hypochlorite solution, followed by repeated rinsing in sterile distilled water. Sterilized seeds were pre-germinated under dark conditions at 22 °C until the radicle germinated to a length of 1 cm. To reduce the mesophilic microbial population, the substrate (Sunshine Mix #3^®^ peat- and vermiculite-based media, Sun Gro Horticulture Canada Ltd., Vilna, AB, Canada) was heat treated at 70 °C for 3 d. Once cooled to 25 °C (room temperature), the substrate was inoculated with one of the two *Trichoderma* formulations, either RS or RSP, by adding it following the manufacturer’s recommended rates, then thoroughly mixed to uniformly distribute the inoculum in the substrate. The *Trichoderma*-treated medium was incubated for 48 h before filling the pots. One set of pots containing the *Trichoderma*-treated substrate was infected with *A. euteiches* while the other set remained untreated (control). Pre-germinated seeds were planted in 10 × 10 cm plastic pots filled with the treated substrate. Plants were grown in the Controlled Environment Facility at the University of Saskatchewan, College of Agriculture and Bioresources building for a period of 4 weeks under controlled conditions in a growth chamber maintained at 22 °C day/16 °C night temperature and 16 h daylight length. Pots were re-randomized every alternate day throughout the experiment to minimize environmental variation. Water was provided as required, and fertilization was carried out weekly with half-strength Hoagland’s nutrient solution (Hoagland and Arnon 1950). The experiment was organized in randomized complete block design with eight lentil genotypes, two *Trichoderma* treatments, two *A. euteiches* infection levels, and four treatment replicates, making a total of 128 pots, each containing three plants per pot.

### 2.3. Aphanomyces euteiches Root Infection

Zoospores of *Aphanomyces euteiches* were produced following a protocol developed in the Plant Pathology Laboratory, University of Saskatchewan, Canada (S. Banniza, pers. comm.). Plants were inoculated with a 5 mL zoospore suspension (concentration 5 × 10^3^ zoospores per mL sterile deionized water) of the root rot pathogen 10 d after transplanting by watering at the base of the plants.

### 2.4. Assessment of Plant Growth

Plants were uprooted 4 weeks after transplanting and the media adhering to roots was removed by vigorous shaking and the roots were rinsed in tap water. Plants were cut at the soil level to separate the shoot from the roots, then the root and shoot lengths were measured for every plant. The root material of single individual plants from each pot was collected and stored at −80 °C until the DNA was extracted. Above- and below-ground plant material of the two other plants in each pot were collected, oven dried at 65 °C for 72 h, and weighed to record the dry weight.

### 2.5. Molecular Identification of Trichoderma Species by Barcoding

Root material from each plant sample was lyophilized in a microcentrifuge tube, then finely powdered by vigorous shaking using a tungsten bead for 2–3 min. Total DNA was extracted from 50 mg of this powdered root material for each sample using Qiagen plant DNeasy^®^ kit following the manufacturer’s instructions. A two-step PCR amplification approach was used to generate ready-to-pool amplicon libraries. In the first PCR, the internal transcribed spacer (ITS) region of the template DNA was amplified using ITS1F (CTTGGTCATTTAGAGGAAGTAA) and ITS2 GCTGCGTTCTTCATCGATGC) primer sets (White et al., 1990; Gardes and Bruns, 1993).

The PCR mixture contained 2.5 µL of 1× Qiagen PCR buffer, 0.5 µL of 25 mM MgCl_2_, 0.25 µL of 10 mM dNTP, 0.2 µL of 5 U/µL Qiagen Taq, 10.55 µL of ultrapure H_2_O, 5 µL ITS1F, 5 µL ITS2 (3 µM solutions), and 1 µL of 1:100 dilution template DNA. The PCR conditions were 96 °C for 10 min; 35 cycles of 1 min at 95 °C (denaturation), 52 °C for 1 min (annealing), and 72 °C for 1 min (elongation); followed by 10 min at 72 °C. The second PCR amplification was used to incorporate the Illumina barcodes. Barcode primers included PE1_CS1 (AATGATACGGCGACCACCGAGATCT ACACTGACGACATGGTTCTACA) and FLD CAAGCAGAAGACGGCATACGAGAT-10 bases barcode sequence—TACGGTAGCAGAGACTTGGTCT. The PCR mixture contained 2 µL of 10× buffer, 3.6 µL of 25 mM MgCl_2_, 1 µL of dimethyl sulfoxide, 0.4 µL of 10 mM dNTP, 0.1 µL of 5 U/µL Qiagen Taq, 9.9 µL of ultrapure H_2_O, 2 µL of 2 µM Illumina barcode, and 1 µL of the first PCR products. The PCR conditions were 95 °C for 10 min; 15 cycles for 15 s at 95 °C (denaturation), 60 °C for 30 s (annealing), and 72 °C for 1 min (elongation); followed by 3 min at 72 °C. The amplification products were purified using the AMPure PCR purification kit (Agencourt Bioscience, Beverly, MA, USA). The Illumina MiSeq (San Francisco, CA, USA) paired-end sequencing was performed by Génome Québec according to the manufacturer’s instructions (McGill University, Innovation Centre, Montreal, QC, Canada).

### 2.6. Bioinformatic Analysis

In total, 10,160,550 ITS sequence reads (NCBI accession = SRR7149016 − SRR7149127) were obtained (average quality = 33). The raw sequences were processed using DADA2 ITS Pipeline Workflow (1.8) (https://benjjneb.github.io/dada2/ITS_workflow.html) in R (v.3.5.0). Sequences containing ambiguous base pairs, long homopolymers (8 bp), low-quality reads (average score < 30), short reads (less than 100 bp), chimeric sequences, singletons and doubletons were excluded. Single nucleotide variants (SNVs) were classified using UNITE fungal database (v. 01.12.2017). The number of reads for each SNV was obtained for each sample. The relative abundance of each SNV was calculated in each sample based on a 0 to 100 scale. SNVs classified as *T. harzianum* and *T. virens* were used to evaluate these two strains in each sample. The relative abundance of genus *Trichoderma* in RS and RSP treatments, and the proportion of *T. harzianum* and *T. virens* in *Aphanomyces* infected and non-infected plants were calculated for each genotype.

### 2.7. In Vitro Biocontrol Assay

In vitro antagonistic activity of *Trichoderma harzianum* strain T-22 was tested against *Aphanomyces euteiches* (AE1) using a plate culture assay adopted from Gupta et al., 2001. A 5 mm agar disc of five-day old culture of *A. euteiches* and *T. harzianum* were placed opposite to one another on water agar plates, 2 cm from the border. Control plates received an agar plug in place of *Trichoderma* strain. Plates were incubated at 25 ± 2 °C for five days and the inhibition of the radial growth of the pathogen was measured. Each treatment was replicated three times and the experiment was repeated twice. The colony diameter of AE1 was measured in the test plates with *Trichoderma* as well as in the control plates and the percentage inhibition of *A. euteiches* by *T. harzianum* over the control was calculated by using the formula given by Vincent (1947) as follows: I = (C − T) × 100/C, where I = percent inhibition of mycelium; C = growth of mycelium in the control, T = growth of mycelium in the treatment [41].

### 2.8. Statistical Analysis

Zeros in the SNV dataset were replaced using a Bayes–Laplace approach using the zCompositions v. 1.2.0 R package and then the data was a centered-log ratio transformed using the CoDaSeq v. 0.99.3 package. Permutation-based multivariate analysis of variance (PERMANOVA) was used to test the significance of the effect of genotype and infection on the relative abundance of fungal communities and *Trichoderma* species in the roots of lentil in R v. 3.5.1 (package vegan). A *p*-value of 0.05 was used as the threshold below which the null hypothesis was rejected. Spearman correlations between the abundance of *Trichoderma* and both the above- and below-ground biomass were made in R v. 3.5.1. A t-test was used to compare the ratios of the abundance of *Trichoderma* species in infected/uninfected plants. An analysis of variance was used to test the effect of the plant genotype, infection by *Aphanomyces euteiches*, and inoculation by *Trichoderma* sp. on root length in lentil.

## 3. Results

**Figure 1:**
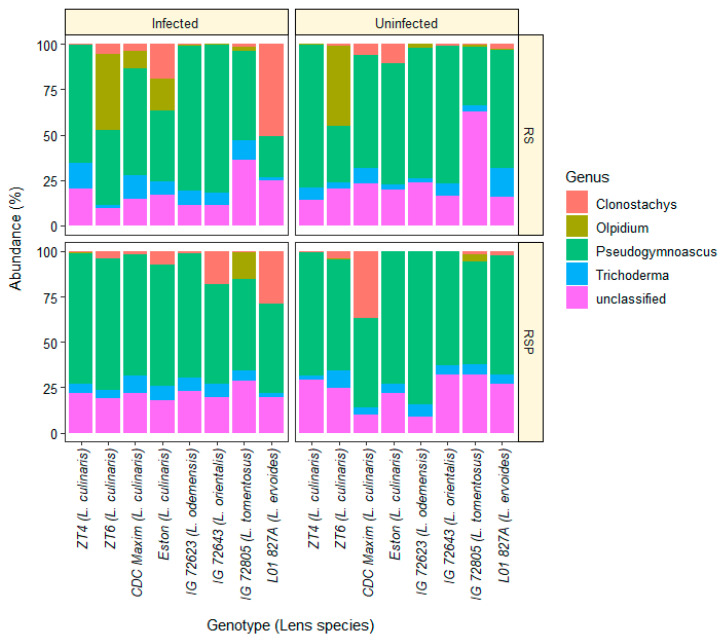
Relative abundance of fungal genera in the roots of diverse lentil genotypes in uninfected conditions (control) or when infected with the pathogen *Aphanomyces euteiches*, four weeks after seeding. The RootShield (RS) product contains *T. harzianum* T-22 and RootShield Plus (RSP) includes *T. harzianum* T-22 and *T. virens* strain G41.

In total, 2441 unique fungal SNVs were detected representing a diversity of fungal taxonomic groups. The four most abundant taxa at the genus level included *Clonostachys*, *Olpidium*, *Pseudogymnoascus*, and *Trichoderma*. Composition of the fungal genera isolated from the roots of lentil genotypes treated with the two *Trichoderma* formulations, *T. harzianum* and *T. virens*, and *A. euteiches* infection at genus level is shown in Figure 1. Furthermore, eight SNVs were classified as *T. harzianum* and four SNVs as *T. virens*, which were used to evaluate these two strains in each sample. Dynamics of the *Trichoderma* SNVs was assessed in different genotypes, treatments, and infection conditions.

### 3.1. Composition of Fungal Communites and Trichoderma Abundance in the Roots

**Figure 2:**
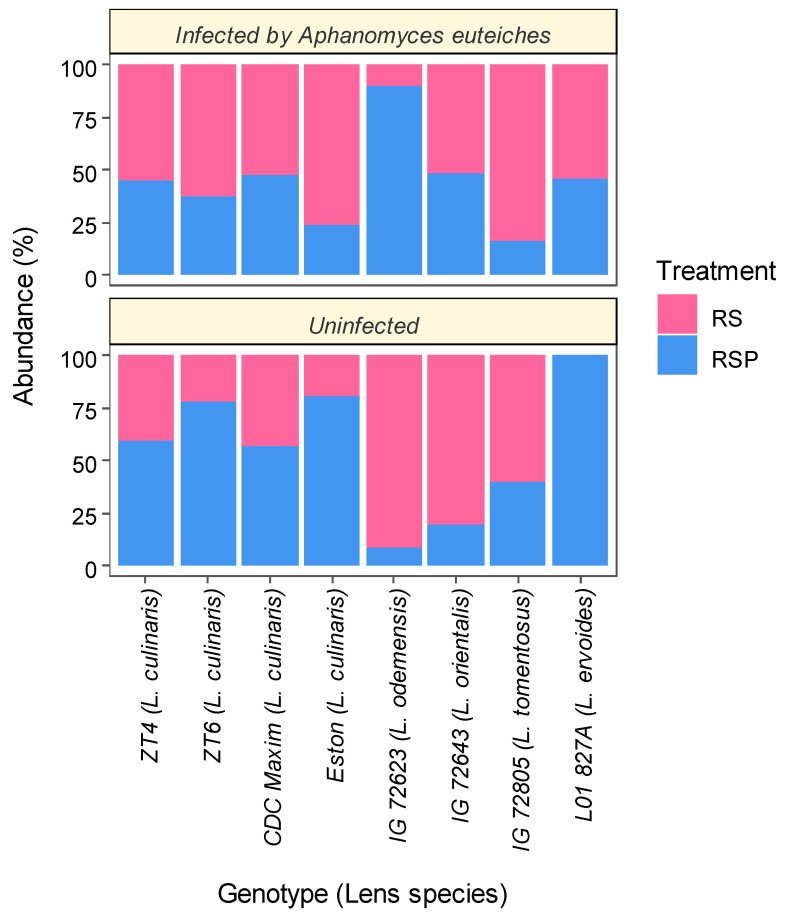
Relative abundance of *Trichoderma* at the genus level in the roots of diverse lentil genotypes treated with one of the two commercial biological products, in uninfected conditions (control) or when infected with the pathogen *Aphanomyces euteiches*, four weeks after seeding. The RootShield (RS) product contains *T. harzianum* T-22 and RootShield Plus (RSP) includes *T. harzianum* T-22 and *T. virens* strain G41. Significant effect of treatment on the abundance of *Trichoderma* was observed for both uninfected and infected plants with *A. euteiches* (*p* < 0.05).

The lentil genotype (*p* < 0.05) and inoculation with the different *Trichoderma* products (*p* < 0.001) significantly influenced the composition of the fungal communities found in the roots (Table 2). The effect of infection of the roots with *A. euteiches* on the composition of the root fungal communities was not significant (*p* = 0.09) (Table 2). Both the lentil genotype (*p* < 0.05) and the *Trichoderma* treatments had significant effects on the composition of *Trichoderma* species in lentil roots (*p* < 0.01) (Table 2). The relative abundance of *Trichoderma* significantly varied between species (*p* < 0.05) (Figure 2).

The relative abundance of *Trichoderma*, at the taxonomic level of the genus, was determined in the root samples collected from all eight lentil genotypes, and accounted for both the endogenous populations of the fungi as well as the inoculation with the *Trichoderma* commercial product strains. In the *Aphanomyces* non-infected plants, the product (RSP) containing both *T. harzianum* T-22 and *T. virens* G41 was the only formulation found to colonize genotype *L. ervoides* L01-827A (100%) (Figure 2). *L. culinaris* genotypes Eston and ZT6 were prevalently colonized by the RSP formulation with both *Trichoderma* spp. In *L. orientalis* and *L. odemensis* the abundance of these two *Trichoderma* from the RSP product was minimal, whereas the RS formulation, containing only *T. harzianum* T-22, highly colonized these two genotypes (Figure 2). When the lentil plants were infected with the ARR pathogen, the abundance of the *Trichoderma* from the single strain RS formulation was greatly modified in all genotypes. In particular, a notable increase in strain T22 was found in Eston and L01-827A in the presence of ARR in comparison to the untreated condition. In contrast, the abundance of *Trichoderma* with the dual strains (RSP) was increased only in the pathogen-infected roots of IG72623 and IG72643 (Figure 2).

**Figure 3:**
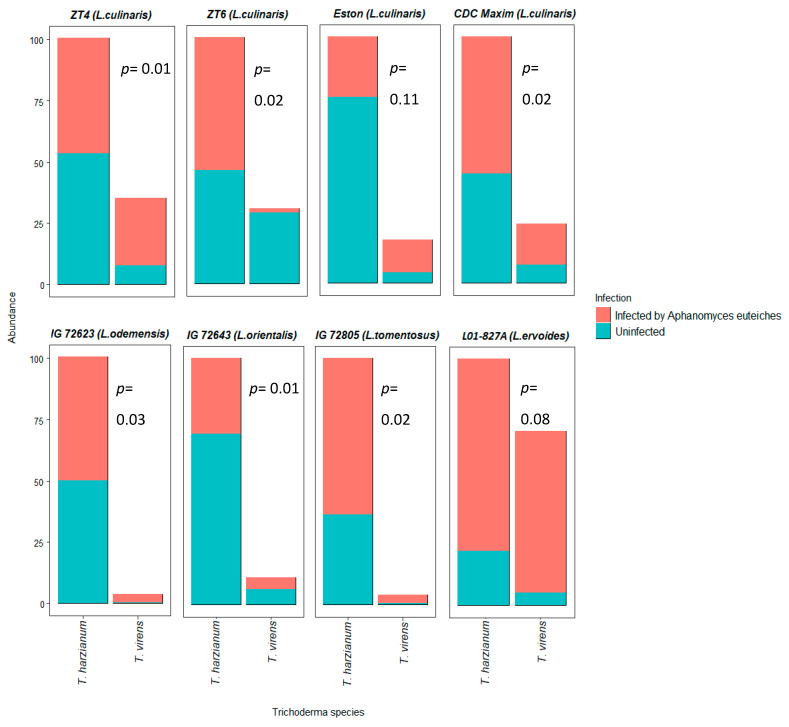
The relative abundance of *T. harzianum* and *T. virens*, as determined by the identification of *Trichoderma* species found in the roots after inoculation with commercial RS or RSP products to each of the eight different lentil genotypes, when grown in uninfected conditions (control) or infected with the pathogen *Aphanomyces euteiches* (AE), four weeks after seeding. Relative abundance of *Trichoderma* significantly varied between species (*p* < 0.05). Statistics presented are the comparison of the uninfected/infected ratios between *Trichoderma* species for each genotype.

Single nucleotide variants, using the selected fungal ITS primers, were able to distinguish and classify the RS and RSP components of *T. harzianum and T. virens. T. harzianum* was always more abundant than *T. virens* in both the healthy and infected roots of all *Lens* genotypes (Figure 3). In the absence of disease, *T. virens* was more abundant in the ZT6 than in the other genotypes. Interestingly, when the plant was infected by *A. euteiches, Lens ervoides* L01-827A showed a unique and significant increase in the abundance of both *Trichoderma* species, but in particular, there was a greater increase in *T. virens* with the pathogen. The abundance of *T. harzianum* was similar in uninfected and *A. euteiches* infected roots in *L. culinaris* genotypes ZT4, ZT6, CDC Maxim and *L. odemensis* IG72623. *T. harzianum* T22 was less abundant in the infected roots of *Lens culinaris* genotypes Eston and *L. orientalis* IG72643, whereas *T. virens* was much less abundant in the infected plants of *L. culinaris* ZT6 than in any of the other infected genotypes. The presence of *T. virens* was the least in *L. odemensis* IG 72623 and *L. tomentosus* IG 70805 of all eight lentil genotypes (Figure 3).

### 3.2. Relationship between Relative Abundance of Trichoderma spp. and Plant Growth

Highly significant effects of lentil genotype and *Trichoderma* treatments on root length (*p* < 0.001) were observed (Table 3). Significantly positive correlations (*p* < 0.05) were observed between the abundance of the *Trichoderma* spp. and the plant growth, both in the above and below-ground vegetative biomass (Table 4).

**Figure 4:**
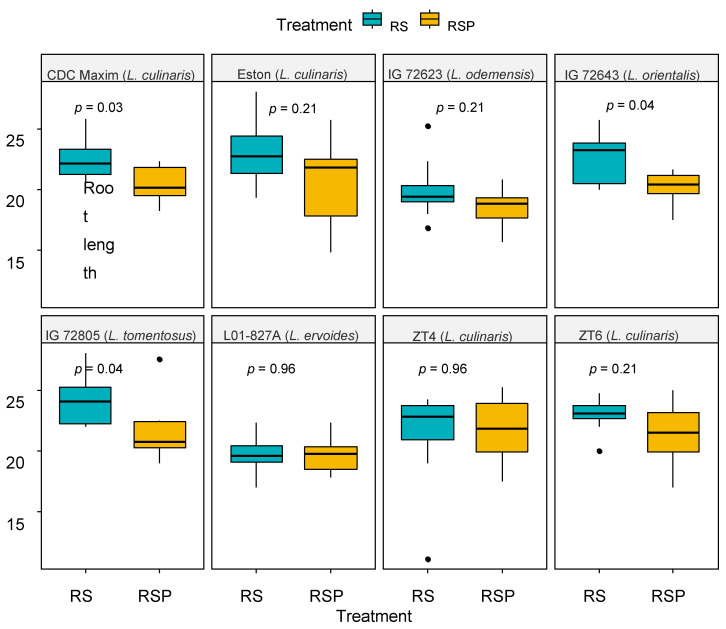
Effect of treatments with two *Trichoderma* products on the root development of lentil genotypes 4 weeks after seeding. RootShield (RS; green boxes) product contains *T. harzianum* T22 and RootShield Plus (RSP; yellow boxes) includes *T. harzianum* T22 and *T. virens* strain G41; the presence of the *A. euteiches* (A) pathogen. The boxes contain the values for all eight genotypes, and the line represents the mean root length value (*n* = 32). The p-values compare the root length effects by RS and RSP in each genotype.

A greater increase in root length was observed in the plants treated with RS in comparison to those treated with RSP (*p* < 0.05), for example, as noted in genotypes CDC Maxim (*L. culinaris*), IG 7263 (*L. orientalis*) and IG 72 805 (*L. tomentosus*) (Table 4 and Figure 4). There was not a strong correlation observed between the growth of the lentil plants’ above- and below-ground parts, and the treatments with the different commercial product tested in the study (Table 4).

### 3.3. In Vitro Biocontrol Plate Assay

**Figure 5:**
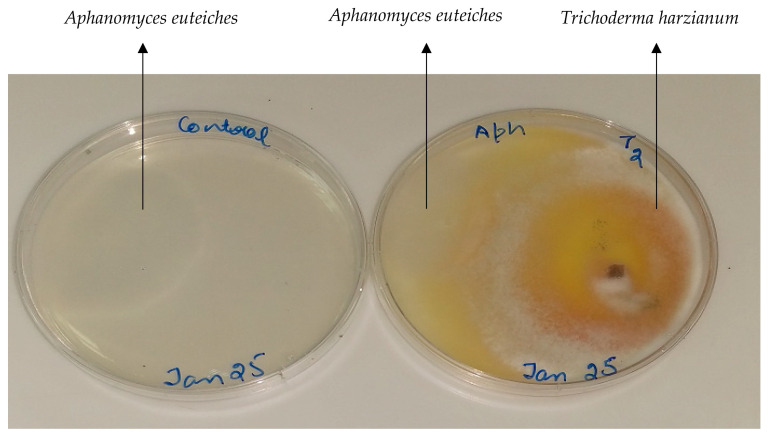
In vitro cultures of: **left**—*Aphanomyces euteiches* alone; **right**—bioassay of *Aphanomyces euteiches* and *Trichoderma harzianum* strain T-22 co-cultures. Five day old culture of *A. euteiches* and *T. harzianum* were used for the assay on water agar plates. Microbial growth showed is after 5 days of incubation.

The average radial diameter of mycelial growth of *A. euteiches* (AE1) alone in control plates was 4.40 ± 0.1 cm while it was only 2.29 ± 0.13 cm in the confrontation bioassays with *T. harzianum* T-22, resulting in a 48% inhibition of the pathogen under in vitro test conditions (Figure 5).

## 4. Discussion

*Trichoderma* species are widely used in agriculture as biopesticides and plant growth stimulants [36,42,43,44,45,46,47,48]. However, the effectiveness of *Trichoderma* products can be inconsistent under variable field conditions [49]. Here, we demonstrated that *T. harzianum* T-22 was capable of inhibiting *A. euteiches* in vitro. The inoculation of *Trichoderma* species, *T. harzianum* and *T. virens,* improved the biomass and root length of *A. euteiches*-infected plants in controlled environmental conditions. However, it did not reduce ARR infection in all eight lentil genotypes tested. The results of many years of research have revealed that not all *Trichoderma* strains exhibiting biocontrol activity in vitro will have the key characteristics required for adaptation to specific environmental conditions, or they may not function well in the conditions in which the plant pathogens flourish [50,51]. Competition between microorganisms for nutrients and space in the rhizosphere, and the possible degradation of useful enzymatic or antibiotic products by other soil microorganisms, may affect the establishment of both *Trichoderma* and *A. euteiches* [52].

The variation in biocontrol efficacy due to substrate properties was shown in pea, whereby *A. euteiches* root rot was significantly reduced by *T. harzianum* alone, however, upon the addition of a soil amendment the biocontrol efficacy was reduced. In fact, physiochemical properties of the substrate can be more favorable to *A. euteiches* growth and development instead of to *Trichoderma* species, thereby reducing the biocontrol efficacy of the *Trichoderma* products [49].

The principal mechanisms employed by *Trichoderma* for biological control include parasitism, the production of secondary metabolites with antibiosis properties, and the induction of plant defense, which can occur singly, simultaneously, or sequentially. However, not all *Trichoderma* strains use all of the abovementioned mechanisms [53]. *Trichoderma* can efficiently induce systemic and localized resistance in plants, trigger a range of responses, including the production of antimicrobial compounds, the deposition of callose on the inner surface of cell walls, and increase chitinase and peroxidase activities. In addition, other plant-beneficial effects by *Trichoderma* also include plant growth promotion. In fact, the secondary metabolites produced by *Trichoderma* have the ability to stimulate plant tissue growth as well as directly act against a range of pathogens [38]. The function of these mechanisms can be influenced by various conditions: soil type, temperature, pH, and moisture of the plant and soil environment and other microorganisms [51]. Therefore, the efficacy of the inoculants is the effect of the multipartite interactions among host plant, *Trichoderma* strains, pathogens and environment [11,52]. As a legume, lentil establishes a symbiosis with N_2_-fixing rhizobia [54]. The synergistic effects of *Trichoderma* and rhizobial species on disease control have been reported [55]. *T. harzianum* and *T. virens* are both aggressive parasites of various pathogenic fungi, and are effective in the stimulation of plant defense responses [56]. In order to minimize the effect of other microorganisms on *Trichoderma* activity, the plants were not inoculated with rhizobia in our experiment, also since two different species of *Trichoderma* were used, and varying, contrasting effects could be potentially expected.

This study investigated and documented plant genotype influence on the specific composition of the fungal community and *Trichoderma* strains colonizing the lentil roots, which was further influenced by the presence or absence of ARR. A previous study reported a genotype-specific influence of the same two *Trichoderma* strains on the agronomic performance of lentil genotypes under *A. euteiches*-infected and uninfected conditions [57]. Results indicated that despite the root colonization of all treated plants, the effect of *Trichoderma* treatments varied significantly, ranging from positive to negative, favorable to unfavorable, differentially effecting above and below-ground plant growth parameters of the lentil genotypes [57]. This suggested that there may be differences in the *Trichoderma* root population structure among the various lentil genotypes, as well as diverse responses in the presence or absence of *A. euteiches*. In our present study, the results clearly indicated that the root colonization affinity by the *Trichoderma* strains varies according to the association with the different lentil genotypes. Moreover, the infection of the *A. euteiches* pathogen is not only influenced by both the lentil genotype and *Trichoderma* species applied, but it subsequently affected the composition of the *Trichoderma* root population. Therefore, the multipartite interactions among the lentil, *Trichoderma* and the *A. euteiches* produces a microbiome that is unique for each lentil genotype and the *Trichoderma* species applied. Furthermore, it was observed that there was an effect on the diversity of root-associated fungi other than the *Trichoderma* inoculated onto the plants, even when the soil/growth media was pasteurized. This suggests that it was probable that the root-associated microbiome could be inherited from the seed microbiome. According to recent reports, the seed microbiome may vary by plant genotype and environmental conditions [58]. The effect of the inherited plant microbiome with treatments of commercial inoculants is yet to be explored. *Trichoderma* is a very common soil fungus and *Trichoderma* endophytes can be inherited from the indigenous pool. However, this study did not identify the indigenous taxa in the root endosphere since the substrate was pasteurized and could not reflect the true indigenous community.

The greater abundance of the *Trichoderma* species in *A. euteiches*-infected conditions in some *Trichoderma*-lentil genotype combinations might be due to increased nutrient and carbon availability, from the release of compounds as a result of root tissue damaged by the pathogen attack [44] or due to the mechanisms that may be selective for beneficial endophytes in the presence of root pathogens [22]. On the other hand, reduced *Trichoderma* populations under pathogen-infected conditions might be attributed to competition between the pathogen and *Trichoderma* for soil nutrients, growth factors, and space [37]. However, the different trends recorded for the same *Trichoderma* strain but in different lentil genotypes, as well as for two different *Trichoderma* strains on the same lentil genotype, indicate that other factors, in addition to nutrient availability and competition, govern the multipartite relationship established between the plant, pathogen, and *Trichoderma*. In particular, plant roots have the ability to modulate the microbial community in the rhizosphere, primarily through the release of root exudates, comprised of sugars, amino acids, proteins, secondary metabolites, and hormones [18], that account for the nutrient availability for the plant and microbiota [59] which mediate the chemical communication between the roots and root-colonizing microbes [60]. For example, phenolic compounds are identified as a major secondary metabolites released by plants [61], which also influence the soil microbiome [62,63]. We recently investigated the root polyphenol composition of four lentil genotypes that were a subset of the eight genotypes used in this study, and observed the genotypic variations in the qualitative and quantitative aspects of root polyphenols, and the influence of *A. euteiches* infection on their composition [64]. Lentil roots release polyphenols in their root exudates at a very early stage of growth, i.e., immediately after planting in soil [65], indicating a selective force with respect to the interaction of rhizosphere microbes and the root environment that plays a role in the establishment of the association between roots and microbes.

Recent reports have revealed that the plant genotype is a key determinant of root microbiota composition for fungi [14,66] as well as bacteria [15,67]. The root colonization by *Trichoderma*, as observed with two other fungal endophytes, involves complex chemical communication and exhibits host-genotype preferences [39]. Furthermore, variations have also been reported among different *Trichoderma* strains [39] utilized, in addition to the host genotypes of the crops to which they are applied, such as maize [53] and tomato [68], in terms of the plant beneficial effects observed from the treatment with *Trichoderma*. The results of this study clearly indicated that each lentil genotype offers a unique environment for the establishment of diverse *Trichoderma* strains, leading to variations in the interactions to each other, as well as with the pathogen. This partially explains the variable effects of *Trichoderma* treatments in different lentil genotypes noted in a previous study [57]. Our results suggested that the colonization abilities of lentil by *Trichoderma* is not only *Lens* species-specific, but also intraspecific to the varietal genetic lines of the species, however, the greatest differences in the fungal colonization were noted between the wild and cultivated lines. In terms of the symbiosis potential of *Trichoderma* spp.-*Lens* genotypes, the modern lentil varieties appeared less promising than the wild varieties. Many components that contribute to the plant host-microbe compatibility, such as the production of various phytochemicals, could be inadvertently removed or enhanced through breeding programs, since the genetic lines have been selected for improved yield and resistance to multiple diseases [69], thus this inadvertently has had a negative affect on the *Trichoderma*–plant interaction. All four *L. culinaris* lines responded similarly to *Trichoderma* inoculation in healthy and infected root conditions, whereas other genotypes showed a wider range of responses (Figure 2). *Lens ervoides,* which represented the tertiary gene pool of lentil [40], showed the highest abundance of both *T. harzianum* and *T. virens*, suggesting that other genotypes may lack the specific genes that regulate the compatible associations with *Trichoderma.* Interspecific hybridization between *Lens ervoides* genotype L01-827A and other *Lens* species may provide opportunities for the introgression of genes that are involved in the recruitment of diverse *Trichoderma* species. In this case, the detection, and the incorporation of those genes in the breeding program may enhance the ability of lentil to better utilize the potentials of *Trichoderma* inoculant when conditions are conducive to *Aphanomyces* infection in the field.

We observed a positive correlation between relative *Trichoderma* abundance and above- and below-ground dry biomass for all genotypes. A significant correlation was also observed between root and shoot ratio biomass, indicating that healthy roots promote above-ground plant growth, as would be expected. Furthermore, significant variations were recorded among the lentil genotypes for all plant growth parameters. Moreover, the *Trichoderma* formulations tested had varied effects on root length, with longer root lengths in RS-treated plants than RSP-treated plants. This confirmed that all *Trichoderma* strains were not equally beneficial for promoting plant growth at least at certain conditions [39], and may even neutralize the positive effect of each other or have negative effects on plant performance [37].

Modern agriculture is highly dependent on the use of chemicals such as pesticides to control plant diseases and fertilizers to enhance production. *Trichoderma*-based products could be an alternative to minimize the negative effects of plant pathogens, however, their performance is largely dependent on the crop plants selected and field conditions. It is notable that *A. euteiches* is presently a ubiquitous pathogen in western Canada, causing extensive root rot disease in legumes. Our results demonstrated that the presence of this pathogen in the rhizosphere influenced the composition of *Trichoderma* species, and consequently, the performance of this beneficial fungus on susceptible crop plants such as lentil. While still significant damage from the pathogen was observed, the application of *Trichoderma* still improved plant performance and diminished the overall negative effects of *A. euteiches*. The genotype-specific plant growth provides a rationale for formulating and developing application strategies to harness the maximum benefits of plant-beneficial fungi and highlights the importance of selecting the compatible host-fungus combination under existing field conditions.

## Figures and Tables

**Table 1:** Lentil genotypes tested in the study.

Genotype	Species	Gene Pool *	Type
CDC Maxim	*L. culinaris*	Primary	Cultivated
Eston	*L. culinaris*	Primary	Cultivated
ZT-4	*L. culinaris*	Primary	Cultivated
ZT-6	*L. culinaris*	Primary	Cultivated
IG 72643	*L. orientalis*	Primary	Wild
IG 72805	*L. tomentosus*	Primary	Wild
IG 72623	*L. odemensis*	Secondary	Wild
L01-827A	*L. ervoides*	Tertiary	Wild

* According to Wong et al. (2015) [40].

**Table 2:** Effect of the lentil genotype, infection by *Aphanomyces euteiches*, and inoculation by *Trichoderma* sp. on the composition of the fungal community and *Trichoderma* community (species) in the lentil roots, determined by a permutation-based multivariate analysis of variance.

	DF	*p*	
		Fungal Community	*Trichoderma* Community
Genotype	7	0.03 *	0.02 *
Infection (*A.s euteiches*)	1	0.09	0.79
Treatment (*Trichoderma*)	1	0.001 ***	0.002 **
Infection × Genotype × Treatment	7	0.01 *	0.35
Residuals	96		

* *p* < 0.05, ** *p* < 0.01, *** *p* < 0.001.

**Table 3:** Effect of the lentil genotype, infection by *Aphanomyces euteiches*, and inoculation by *Trichoderma* sp. on root length in lentils determined by the analysis of variance.

	DF	Sum Sq	Mean Sq	F	*p*
Genotype	7	167.2	23.89	3.66	0.001 **
Infection (*A. euteiches*)	1	0.1	0.07	0.01	0.91
Treatment (*Trichoderma*)	1	72.1	72.1	11.07	0.001 **
Infection × Genotype × Treatment	7	26.5	3.79	0.58	0.76
Residuals	96				

** *p* < 0.01.

**Table 4:** Relationship between the lentil root biomass or shoot biomass to the relative abundance of *Trichoderma* spp. and between the vegetative below- and above-ground biomass, as indicated by Spearman’s correlation.

Factors	*p* Value	r
Root biomass: *Trichoderma* spp.	<0.05	0.2
Shoot biomass: *Trichoderma* spp.	<0.05	0.19
Root biomass: shoot biomass	<0.0001	0.51
